# Active and latent tuberculosis among HIV-positive injecting drug users in Indonesia

**DOI:** 10.7448/IAS.18.1.19317

**Published:** 2015-02-16

**Authors:** Hinta Meijerink, Rudi Wisaksana, Mery Lestari, Intan Meilana, Lydia Chaidir, Andre JAM van der Ven, Bachti Alisjahbana, Reinout van Crevel

**Affiliations:** 1 Department of Internal Medicine, Radboud University Medical Centre, Nijmegen, The Netherlands; 2 Health Research Unit, Faculty of Medicine, Universitas Padjadjaran/Hasan Sadikin Hospital, Bandung, Indonesia; 3 Department of Internal Medicine, Faculty of Medicine, Hasan Sadikin Hospital, Padjadjaran University, Bandung, Indonesia

**Keywords:** cohort studies, *Mycobacterium tuberculosis*, human immunodeficiency virus, substance abuse, intravenous, latent tuberculosis infection

## Abstract

**Introduction:**

Injecting drug use (IDU) is associated with tuberculosis but few data are available from low-income settings. We examined IDU in relation to active and latent tuberculosis (LTBI) among HIV-positive individuals in Indonesia, which has a high burden of tuberculosis and a rapidly growing HIV epidemic strongly driven by IDU.

**Methods:**

Active tuberculosis was measured prospectively among 1900 consecutive antiretroviral treatment (ART)-naïve adult patients entering care in a clinic in West Java. Prevalence of LTBI was determined cross-sectionally in a subset of 518 ART-experienced patients using an interferon-gamma release assay.

**Results:**

Patients with a history of IDU (53.1%) more often reported a history of tuberculosis treatment (34.8% vs. 21.9%, *p*<0.001), more often received tuberculosis treatment during follow-up (adjusted HR=1.71; 95% CI: 1.25–2.35) and more often had bacteriologically confirmed tuberculosis (OR=1.67; 95% CI: 0.94–2.96). LTBI was equally prevalent among people with and without a history of IDU (29.1 vs. 30.4%, NS). The risk estimates did not change after adjustment for CD4 cell count or ART.

**Conclusions:**

HIV-positive individuals with a history of IDU in Indonesia have more active tuberculosis, with similar rates of LTBI. Within the HIV clinic, LTBI screening and isoniazid preventive therapy may be prioritized to patients with a history of IDU.

## Introduction

Tuberculosis is a major global health problem, ranking as the second leading cause of death from an infectious disease [1]. Individuals who are more often exposed to *Mycobacterium tuberculosis*, such as alcoholics, homeless people and prisoners, develop more tuberculosis [2–4]. However, not everyone who is exposed to *M. tuberculosis* becomes infected, and not all who are infected will develop active tuberculosis [5]. HIV infection and other factors which suppress host immunity increase the risk of progression to active tuberculosis after latent tuberculosis infection (LTBI) [5]. There are also data to suggest that decreased cellular immunity increases the risk of becoming infected after exposure to *M. tuberculosis* [5].

Injecting drug use (IDU) is associated with active tuberculosis, with most studies coming from high-income countries [3, 4, 6–9]. This is usually explained by factors related to higher tuberculosis exposure in shooting galleries, dormitories and prisons [2–4]. HIV infection also contributes to the high tuberculosis burden among injecting drug users. In addition, lower utilization of health services, poor treatment compliance and low completion rates among drug users may lead to more severe or prolonged tuberculosis, and more transmission in this group [2, 10, 11]. However, IDU by itself may be an independent risk factor for active tuberculosis [6–8, 12]. Studies examining the association of IDU with LTBI have yielded mixed results [13–15]. Besides higher exposure to tuberculosis leading to more LTBI, factors related to drug use may also increase the incidence and severity of active tuberculosis. For instance, opioids as well as hepatitis C virus (HCV) co-infection may have immunosuppressive effects [16–20]. This may also explain why drug users more often present with extrapulmonary tuberculosis, and more slowly respond to treatment [2, 10, 21, 22].

So far, epidemiological studies on IDU, HIV and tuberculosis mostly come from high-resource settings with relative low tuberculosis rates. In addition, few studies have looked at the relation of IDU with both latent and active tuberculosis. Therefore, we examined the association of IDU with the prevalence of LTBI and incidence of active tuberculosis among HIV-positive individuals in Indonesia, which has a high tuberculosis burden and a rapidly growing HIV epidemic strongly driven by IDU [23].

## Methods

### Study population and setting

This study was embedded in a programme called Integrated Management of Prevention and Care and Treatment of HIV/AIDS (IMPACT) aimed to improve prevention, control and treatment of HIV in the context of IDU in West Java, Indonesia. IMPACT has helped establish patient care in the clinic of the referral hospital in Bandung, the capital of West Java (40 million people). In this clinic, people with a history of injecting drugs or high sexual risk behaviour are undergoing voluntary counselling and testing [24]. In addition, patients may be referred for HIV testing if they present with signs and symptoms suggesting HIV/AIDS, such as oral candidiasis, chronic diarrhoea, tuberculosis and hepatitis C infection. All testing is voluntary and informed consent is obtained from all study participants. HIV-positive patients are characterized and followed prospectively in a cohort study, which has been approved by the Health Research Ethics Committee at the Faculty of Medicine of Padjadjaran University/Dr. Hasan Sadikin General Hospital in Bandung, Indonesia. Data on demographic factors, history of IDU, co-morbidity, self-reported tuberculosis treatment and history of antiretroviral treatment (ART) are collected through interviews with standard questionnaires. A history of IDU was defined as “ever injecting drugs” without differentiating previous or current drug users. Patients are extremely reluctant to discuss current drug use because it is highly criminalized in Indonesia. Laboratory examinations include CD4 cell measurement at baseline and regularly afterwards. Patients were seen by a doctor every month or if they had clinical symptoms, during which time they were examined for oral thrush and tuberculosis-suggestive symptoms. ART is indicated in Indonesia for patients presenting with WHO Stage IV or a CD4 count less than 350 cells/µl, in accordance with WHO guidelines from 2006. Since 2004, ART can be accessed free of charge in Indonesia.

### Cohort study: active tuberculosis

**Figure 1:**
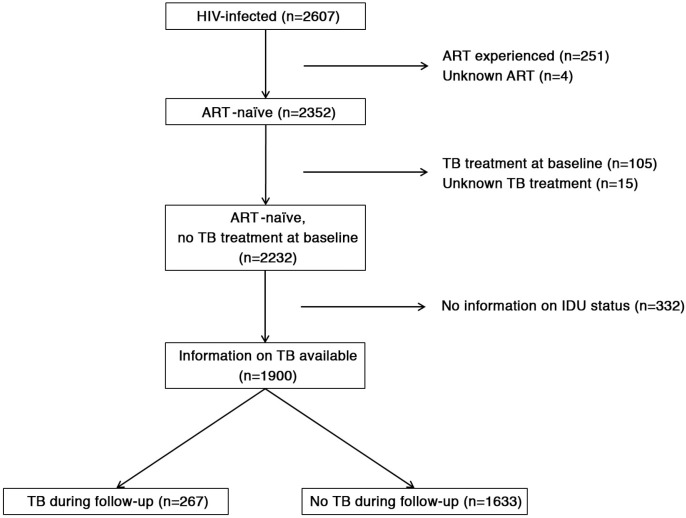
HIV-positive subjects who were ART-naïve and treatment-naïve for TB were followed prospectively and were compared according to tuberculosis treatment status during follow-up.

To determine the association between IDU and the incidence of HIV-associated tuberculosis, we used data collected at the HIV clinic. For this study, we selected all adults (≥16 years) presenting with HIV infection between August 2007 and February 2013, and who were ART-naïve at time of first presentation (Figure 1). Follow-up data were included up to August 2013. Patients were censored either at their last visit or on 1 August 2013. Time of follow-up was considered zero for all patients enrolled without a follow-up visit. We used tuberculosis treatment as an indicator for active tuberculosis, because the diagnosis of active tuberculosis and decision to start tuberculosis treatment was often based on clinical symptoms and chest X-ray. During clinic visits, patients were asked whether they were taking tuberculosis treatment, and prescription of tuberculosis treatment by the doctors in the HIV clinic was recorded. We also analyzed data from all HIV-positive patients with sputum examination results. Sputum was collected in the HIV clinic and examined using Ziehl–Nielsen or fluorescence microscopy and *M. tuberculosis* culture using solid media or MODS [25]. Bacteriologically confirmed tuberculosis was defined as positive microscopy (either Ziehl–Nielsen or fluorescence microscopy) or *M. tuberculosis* culture (either solid media or MODS). All patients who already received tuberculosis treatment at baseline were excluded from this analysis (Figure 1).

### Cross-sectional study: LTBI

Testing for LTBI has not been implemented in Indonesia. The first priority in HIV care is to exclude active tuberculosis before or shortly after starting of ART (“unmasking tuberculosis”). For the purpose of this study, LTBI was examined in a cross-sectional survey among HIV-positive patients visiting the HIV clinic between April 2012 and April 2013. Patients receiving ART for less than three months were excluded, as the CD4 cell count for those patients are often very low in this population [23]. After informed consent, all patients were interviewed and examined. BCG vaccination was based on visual confirmation of a scar and self-report; oral thrush was diagnosed by the physician. Exposure to tuberculosis inside and outside the household was based on self-reported contact with individuals receiving tuberculosis treatment.

For LTBI screening, we used an interferon-gamma release assay, the Quantiferon Gold In-Tube assay, according to manufacturer's instructions. Briefly, blood was collected in the provided tubes – nil control, tuberculosis antigen and mitogen control. Tubes were immediately incubated for 16 to 24 hours at 37°C. After centrifugation, we collected the supernatant and stored the samples at −80°C until ELISA could be performed to determine IFN-γ production. Individuals were categorized as positive for LTBI when the tuberculosis antigen minus the nil control was higher than 0.35 IU/ml and over 25% of the nil control. All samples with high nil controls (>8.0 IU/ml) or low mitogen controls (<0.50 IU/ml) were seen as indeterminate.

All patients with LTBI were seen by a doctor and examined for active tuberculosis. If no active tuberculosis was found, patients received isoniazid preventive therapy (IPT) according to WHO guidelines.

### Data analysis and statistics

Individuals with missing data on IDU were excluded from further analysis. Quantitative variables were expressed as median and interquartile ranges (IQR) and categorical variables as frequencies. Differences in baseline characteristics between individuals with and without a history of IDU were examined using the Mann–Whiney test and Chi-square. The effect of IDU on the incidence of HIV-associated tuberculosis was examined using univariate and multivariate Cox proportional hazard regression analyses. Proportionality assumption was verified for all variables using Kaplan–Meier analyses. In addition, the association between IDU and the incidence of tuberculosis was also examined among those without any history of tuberculosis, because rate of reinfection is higher than the rate of new infection [26]. Haemoglobin was used as proxy for nutritional status, because BMI was missing for a large proportion of the study population (20.6%; 391/1900). ART initiation was taken into account as a time-dependent covariate in Cox proportional hazard model, because time on ART is associated with decreased risk of progression to active tuberculosis. Laboratory confirmed tuberculosis was examined with univariate and multivariate logistic regression analyses, because exact time of diagnosis was not always provided.

The LTBI prevalence in relation to IDU was examined using univariate regression analyses. The influence of possible confounders, such as previous active tuberculosis infection, exposure to tuberculosis and CD4 cell counts were explored using multivariate regression analyses.

The variables tested were chosen based on literature and used in both univariate and multivariate analyses [27, 28]. We assessed the fit of all models using −2 log likelihood. All statistical analyses were done using SPSS statistical software (version 20.0).

## Results

In total, 2607 HIV-positive patients were enrolled at the HIV clinic during the study period. Of these, 251 already received ART and 4 individuals had no information about ART use (Figure 1). Information on IDU was available for 1900 individuals; the 332 individuals with missing data did not differ from those with data on IDU (age, *p=*0.186; haemoglobin, *p=*0.123; sex, *p=*0.482; CD4 cell count, *p=*0.165; BMI, *p=*0.805). The majority of HIV-positive patients had a history of IDU (53.1%; 1009/1900). Those with a history of IDU were mostly male (90.6%; 914/1009), often with high education; 33.0% (325/986) had a university degree versus 27.4% (225/821) in the group without a history of IDU. At baseline, individuals with a history of IDU were more likely to present with advanced HIV infection (Table 1), with more infections (such as oral thrush) and lower CD4 cell counts; the median CD4 cell count in this group was 71 cells/µl compared to 157 cells/µl among those without a history of IDU (Table 1). Similarly, WHO Stage IV was present in 49.5% of those with a history of IDU, and 33.7% of those without.

**Table 1:** Characteristics of HIV-positive, treatment-naïve patients with and without a history of injecting drug use (IDU)^a^

	With history of IDU (*n=*1009)	Without history of IDU (*n=*891)	*p*
Oral thrush	40.8 (396/970)	29.8 (246/825)	<0.001
Hepatitis C antibodies	90.2 (599/664)	12.8 (55/429)	<0.001
Mortality during follow-up	8.9 (90/1009)	6.7 (60/891)	0.078
Start ART during follow-up	69.8 (703/1007)	56.7 (505/891)	<0.001
Median CD4 cell count, cells/µl (IQR)	71 (18–249)	157 (31–340)	<0.001
Median haemoglobin level, g/dl (IQR)	13.2 (11.3–14.8)	12.1 (10.4–13.4)	<0.001
Median BMI, kg/m^2^ (IQR)	19.2 (17.3–21.6)	19.9 (17.6–22.7)	0.002
Median follow-up, days (IQR)	686 (83–1575)	237 (24–951)	<0.001
Tuberculosis symptoms and treatment status (%)			
History of tuberculosis treatment	34.8 (332/953)	21.9 (172/784)	<0.001
Weight loss (>10%)	38.4 (382/996)	32.6 (273/837)	0.011
Chronic cough (>3 weeks)	21.3 (212/993)	22.2 (185/835)	0.677
Night sweats	1.6 (16/1009)	2.7 (24/891)	0.093
Fever (>1 week)	28.5 (284/996)	24.6 (206/838)	0.058
At least one of the above symptoms	48.3 (487/1009)	41.9 (373/891)	0.005
Tuberculosis treatment during follow-up^b^	17.5 (177/1009)	10.1 (90/891)	<0.001

a Unless stated otherwise, data are given as percentage (numerator/denominators). Data were missing for CD4 cell count (68 with and 119 without IDU), haemoglobin (78 with and 127 without IDU) and for BMI (188 with and 203 without IDU).

b Individuals receiving tuberculosis treatment either at the HIV clinic or at the hospital based on medical status and pharmacy records. IDU, injecting drug use; ART, antiretroviral treatment; IQR, interquartile range; BMI, body mass index.

We excluded 105 patients who were taking tuberculosis medication at enrolment in the HIV clinic (Figure 1). Compared to those patients included in further analysis, these 105 patients did not differ with regards to history of IDU (*p=*0.876) and age (*p=*0.188), but they were more often male (*p*<0.001) and had lower haemoglobin (<0.001) and lower CD4 cell counts (*p*<0.001). Data on reported previous tuberculosis treatment were available for 1737 out of 1900 patients who were ART-naïve and who were not taking tuberculosis medication at the time of enrolment in the study. One fourth of this group (26.5%) reported previous tuberculosis treatment. HIV-positive patients with a history of IDU significantly (*p*<0.001) more often had a history of tuberculosis treatment (34.8%) than those without a history of IDU (21.9%, Table 1). At baseline, patients with a history of IDU did not have more symptoms suggesting tuberculosis; weight loss and fever were more common but chronic cough and night sweats were not (Table 1). Patients with a history of IDU were more likely to report any of these symptoms, but this difference disappeared after correction for CD4 cell count (adjusted OR=0.92; 95% CI: 0.74–1.15). The same was found when symptoms were analyzed separately (data not shown).

**Figure 2:**
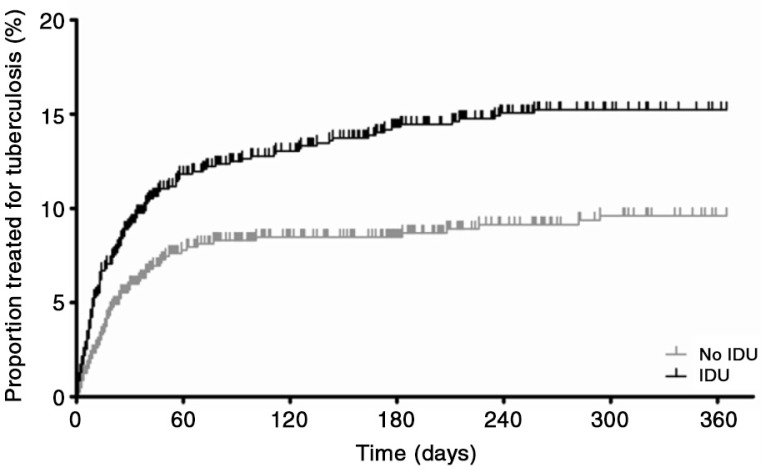
Tuberculosis incidence for ART-naïve, HIV-positive individuals with (black) and without (grey) a history of injecting drug use (IDU) in the first year after enrolment in HIV care (*n=*1,900). Tuberculosis incidence was more common among patients with a history of IDU, also when corrected for age, CD4 cell count, haemoglobin, antiretroviral treatment and oral thrush (adjusted hazard ratio 1.71 with 95% CI: 1.25–2.35).

Among 1900 patients, 267 individuals developed tuberculosis during follow-up (Figure 1). In total, 17.5% of those with and 10.1% of those without a history of IDU received tuberculosis treatment during follow-up (*p*<0.001). Patients with a history of IDU were 1.7 times more likely to receive tuberculosis treatment in the first year after enrolment in HIV care, also after adjustment for possible confounders (Table 2 and Figure 2). Tuberculosis treatment was mostly prescribed in the first few months after enrolment in care. The majority of tuberculosis cases (55.3%) were diagnosed before start of ART, 18.4% within three months of ART, 12.3% after three months of ART, whereas 14.0% of HIV-positive patients treated for tuberculosis did not receive ART during the follow-up period. The higher incidence of tuberculosis among HIV-positive patients with a history of IDU remained significant with longer follow-up, with an adjusted hazard ratio of 1.54 (95% CI: 1.15–2.07) during two years and 1.52 (95% CI: 1.13–2.06) during three years of follow-up after enrolment in HIV care. When excluding everyone with previous tuberculosis, patients with a history of IDU still had significantly more tuberculosis (14.0% vs. 8.0%), with an adjusted hazard ratio of 2.07 (95% CI: 1.30–3.28) for patients with a history of IDU in the first year after enrolment in HIV care.

**Table 2:** Factors associated with active tuberculosis during one year follow-up of ART-naïve patients entering HIV care (*n=*1900)

	Univariate		Multivariate	
		
Factors^a^	HR (95% CI)	*p*	HR (95% CI)	*p*
History of injecting drug use	1.76 (1.33–2.32)	<0.001	1.71 (1.25–2.35)	0.001
CD4 cell count, per 10 cells/µl	0.94 (0.93–0.96)	<0.001	0.97 (0.95–0.98)	<0.001
Age	1.03 (1.01–1.04)	0.007	1.02 (0.99–1.04)	0.199
Haemoglobin, g/dl	0.78 (0.74–0.81)	<0.001	0.83 (0.78–0.88)	<0.001
Oral thrush	3.43 (2.64–4.46)	<0.001	1.66 (1.19–2.29)	0.002
Antiretroviral treatment^b^	1.22 (0.95–1.57)	0.122	0.91 (0.68–1.23)	0.555

a Factors are determined at enrolment in HIV care

b antiretroviral treatment was taken into account as time-dependent variable. Data were missing for CD4 cell count (*n*=108), age (*n=*28), haemoglobin (*n=*205), oral thrush (*n=*105) and ART (*n=*2). For multivariate analyses, all individuals with missing data were excluded (*n=*213). ART, antiretroviral treatment; HR, hazard ratio; CI, confidence interval.

These data were supported by a higher rate of bacteriological confirmed tuberculosis among drug users. From 255 ART-naïve individuals with available data on sputum examination, 115 (45.1%) had a positive sputum microscopy or *M. tuberculosis* culture – 68 out of 143 with a history of IDU (47.6%) and 47 of 112 non-drug users (42.0%). After adjustment for possible confounders (CD4 cell count, haemoglobin and oral thrush based on Table 2), HIV-positive patients with a history of IDU seemed to have a higher rate of bacteriological confirmed tuberculosis (OR=1.67; 95% CI: 0.94–2.96).

**Figure 3:**
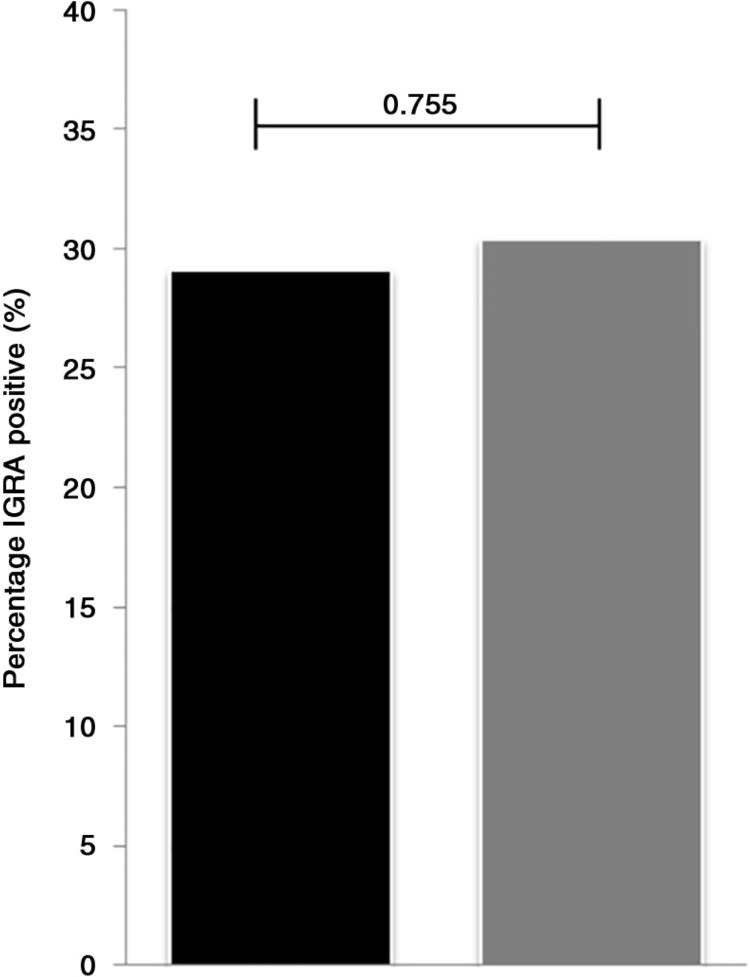
The proportion of HIV-positive individuals with (black) and without (grey) a history of injecting drug use (IDU) who were IGRA positive. Groups were compared using χ^2^ and resulted in a χ^2^ of 0.097 with a *p*-value of 0.755.

To determine if the higher incidence of active tuberculosis could be explained by a higher prevalence of latent tuberculosis, we performed a cross-sectional study on the prevalence of LTBI (Table 3). In total, 524 HIV-positive individuals who were ART experienced (median 41 months; IQR: 26 to 61 months) and who had a median CD4 cell count of 373 (IQR: 268 to 518) were included. Table 3 shows the characteristic of the study population at the time to testing for LTBI. Among them, 29.2% had a positive Quantiferon test; this was not different among patients with and without a history of IDU (28.5 and 30.3%, respectively, Figure 3). Also, after adjustment for age, CD4 cell count, haemoglobin and ART, no association was found between latent tuberculosis and a history of IDU (adjusted OR=0.91; 95% CI: 0.59–1.40; *p*=0.658).

**Table 3:** Characteristics of HIV-positive patients (*n=*524) with and without a history of injecting drug use (IDU) who were tested for latent tuberculosis infection (LTBI)^a^

	With history of IDU (*n=*312)	Without history of IDU (*n=*212)	*p*
Median age, years (IQR)	33 (30–35)	31 (28–36)	0.001
Male	91.3 (285/312)	31.1 (66/212)	<0.001
Married	56.1 (175/312)	55.7 (118/212)	0.923
Oral thrush	3.6 (11/209)	1.9 (4/210)	0.269
Median BMI, kg/m^2^ (IQR)	20.8 (19.2–22.7)	21.4 (19.0–23.4)	0.225
Median CD4 cells, cells/µl (IQR)	373 (269–522)	377 (263–510)	0.588
Median haemoglobin, g/dl (IQR)	14.4 (13.3–15.3)	12.9 (11.7–14.0)	<0.001
Median time on ART, months (IQR)	49 (34–73)	31 (18–48)	<0.001
Tuberculosis-related characteristics^a^			
IGRA positive^b^	28.5 (88/309)	30.3 (63/208)	0.657
BCG vaccination^c^	75.2 (233/310)	78.1 (164/210)	0.440
Current smoking	87.8 (274/312)	34.6 (73/211)	<0.001
History of tuberculosis treatment	46.9 (144/307)	31.4 (66/144)	<0.001
Exposed to tuberculosis in household	9.8 (20/305)	30.0 (61/203)	<0.001
Exposed to tuberculosis outside household	33.2 (80/241)	25.8 (41/159)	0.114

a Unless stated otherwise, data are given as percentage (numerator/denominators). Data were missing for CD4 cell count (5 with and 0 without IDU), haemoglobin (18 with and 17 without IDU) and BMI (5 with and 3 without IDU)

b IGRA positive: interferon-gamma release assay (Quantiferon Gold In-Tube) was used to determine latent tuberculosis infection. Individuals were classified as positive when tuberculosis antigen minus the nil control was higher than 0.35 IU/ml and over 25% of the nil control

c BCG vaccination was based on visual confirmation by a physician or self-reported in case visual confirmation was indeterminate. IDU, injecting drug use; IQR, interquartile range; BMI, body mass index; ART, antiretroviral treatment; IGRA, interferon-gamma release assay.

## Discussion

In this large cohort of patients presenting with advanced HIV infection in Indonesia, those with a history of IDU were more likely to have a history of tuberculosis treatment, to start tuberculosis treatment and to have bacteriological confirmed tuberculosis. The prevalence of LTBI in a subset of ART exposed individuals was not different, which may suggest that HIV-positive patients with a history of IDU have a higher risk of progression to active tuberculosis after *M. tuberculosis* infection.

Our results on active tuberculosis are consistent with those of other studies, which found a two to three times increased risk in active tuberculosis among injecting drug users [6–8, 28]. Yet, these studies took place in high-income countries with low tuberculosis burden. One of the few studies in developing countries is that of Cain *et al*., who evaluated a diagnostic algorithm for tuberculosis among HIV-positive individuals in Cambodia, Thailand and Vietnam [29]. Although not primarily designed to address this question, the prevalence of active tuberculosis was more than three times higher in injecting drug users in this cohort.

Lower access to health services, poor treatment compliance and increased exposure to *M. tuberculosis*, because of homelessness, crowding and incarceration [2–4], increased the tuberculosis risk among drug users. However, we do not expect higher exposure to *M. tuberculosis* among HIV-positive patients with a history of IDU in Indonesia, where interestingly enough, HIV and IDU are often associated with higher education and socio-demographic background [23, 30]. In addition, unlike in Western countries, drug use in Indonesia is not associated with additional risk factors, such as homelessness, unemployment and crowding, as seen in Western countries. This, and the fact that tuberculosis is endemic in Indonesia, implicate that tuberculosis exposure is comparable in people with and without a history of IDU in this setting. In addition, we previously showed that patients with and without a history of IDU receiving ART have similar outcomes with regards to treatment adherence, mortality rates, loss to follow-up rates and virological failure [22].

In this setting, the prevalence of LTBI was similar in ART-experienced HIV patients with and without a history of IDU. Earlier studies comparing the prevalence of LTBI between injecting drugs users and non-users have shown mixed results [15, 31]. We diagnosed LTBI using IGRA, because Indonesia has routine BCG and because IGRA, unlike the Mantoux tuberculin skin test, does not require a return visit. There was possibly selection bias because we did not include patients diagnosed with or presenting with symptoms suggesting tuberculosis. However, we expect this bias to be limited because most cases of tuberculosis were diagnosed in the first period after enrolment in HIV care (Figure 2) and the majority of patients tested for LTBI had received ART for more than two years. Therefore only a few patients were excluded due to diagnosis of active tuberculosis. In addition, we expect that the exposure to tuberculosis is similar among those receiving and those not receiving ART. By only selecting ART-experienced individuals, the risk of false-negative Quantiferon tests is lower, because of reconstituted CD4 cell counts.

Our results provide support for the hypothesis that underlying biological factors (rather than increased exposure and infection rates) contribute to a higher risk of active tuberculosis in injecting drug users. A possible explanation is that opioids affect the immune response directly; *in vitro* and animal studies have found deleterious effects of opioids in infections [20]. In relation to *mycobacteria* specifically, studies show mixed results; some finding advantageous effects of opioids [32], others showing deleterious effects [33, 34]. In addition, we have previously shown biological effects of heroin use on the expression of chemokines and chemokine receptors among HIV-infected individuals [35]. Chronic HCV infection, highly prevalent among injecting drug users, could be another factor. There is no clear association between chronic HCV and HIV disease progression [18], but HCV co-infection can impair cellular immunity, independent from CD4 cell count. Also, HCV infection is known to cause iron overload, which in itself is associated with active tuberculosis [36]. We could not examine an independent effect of HCV in this study because almost all injecting drug users in our setting were positive for HCV [30].

We adjusted our results for CD4 cell count as proxy for immune status in HIV infection. However, the total number of CD4 cells may not correlate well with the number of *M. tuberculosis*-specific CD cells [37, 38]. HIV-positive patients with a history of IDU in this cohort experience a more rapid natural decline in CD4 cells [30], and it might therefore be hypothesized that they have a preferential depletion of these specific *M. tuberculosis* CD4 cells, in which case adjustment for CD4 cells would not be adequate.

Most patients in our cohort were diagnosed with tuberculosis within the first few months after enrolment in HIV care. The majority of individuals start ART after diagnosis of tuberculosis, which suggests that individuals go to the HIV clinic when symptoms of tuberculosis arise. In addition, we show that in 18.4% initiation of ART “unmasked” tuberculosis [39]. In our study, HIV-positive patients with a history of IDU more often report a history of previous tuberculosis treatment, and were more often treated for tuberculosis during follow-up, although rates of latent tuberculosis were similar.

In this study, we used tuberculosis treatment as an indicator for active tuberculosis. Ideally, bacteriological examination would be used as endpoint, but unfortunately this was only available for a small proportion of patients. Tuberculosis treatment could be influenced by indication bias; doctors might be more likely to prescribe tuberculosis treatment to people with a history of drug use. However, sensitivity analysis using data from the bacteriological examination showed a similar trend, indicating that probably no bias took place with regard to prescribing tuberculosis treatment. Possibly, overall immune suppression of opioids masks tuberculosis and therefore more tuberculosis is unmasked in people with a history of IDU. Yet, we also see a higher prevalence of previous tuberculosis treatment and therefore it seems that HIV-positive individuals with a history of IDU are at higher risk of progression to active tuberculosis.

In Indonesia, IDU is a major route of transmission and in our cohort 53% of patients had a history of IDU. Even though the majority of new infections worldwide occur through heterosexual contact, IDU is an important transmission route for new infections outside of Africa, for instance East Europe, Southeast Asia and North and South America [40]. In most Asian countries, the national HIV prevalence is relatively low, but the HIV epidemic resides in key populations, such as sex workers, men having sex with men and injecting drug users. A recent report from UNAIDS shows that HIV prevalence among injecting drug users ranged from 6.3 to 56.4% [41]. In three countries in Asia with expanding epidemics – Indonesia, Pakistan and the Philippines – IDU has been a significant factor in the spread of HIV. Cain *et al*. showed that among HIV individuals in Vietnam, Cambodia and Thailand, 14% had a history of IDU [29]. Our results could therefore be important in other regions, where IDU is an important route of transmission and tuberculosis is prevalent.

In this study, we excluded patients with missing data for tuberculosis and/or a history of IDU, and this may have led to selection bias. However, no differences were found between individuals with and without missing data. For the cross-sectional study on LTBI, we selected ART-experienced patients. All patients on ART are required to visit the clinic once a month for a check-up and to receive their medication. Those selected for this study were therefore a representative for HIV-positive individuals receiving ART. However, we should be careful to extrapolate these results to all HIV-positive individuals in Indonesia. Another limitation of the study is the fact that we did not assess the effects of nutritional status, smoking or HIV viral loads as possible (confounding) risk factors for tuberculosis incidence.

Our findings have implications for health policy and practice. First, people with a history of IDU should be actively tested for HIV, and HIV treatment should be started according to WHO guidelines, especially because ART has been shown to protect against development of tuberculosis, irrespective of CD4 cell count [42]. In addition, testing for LTBI and IPT could be prioritized to patients with a history of IDU. Active case finding might be improved by involving local non-government organizations and educating people about the risk when not treated in time. Also screening for active tuberculosis should focus on injecting drug users, although screening is of course advised for all HIV-positive individuals.

## Conclusions

The results from this study suggest that active tuberculosis is more prevalent among HIV-positive individuals with a history of IDU, and that this cannot be explained by higher rates of LTBI. It should be noted that individuals with a history of IDU in our cohort are not from a lower or impoverished background, which might affect disease progression or exposure to tuberculosis; most had higher education and higher social economic background. Instead, we suspect that biological factors related to IDU play a major role, such as a direct effect of opioids, a difference in tropism or virulence of HIV virus transmitted via sharing needles or underlying conditions such as HCV infection. Further research is needed to address these issues.
